# Performance Analysis of Millimeter-Wave Multi-hop Machine-to-Machine Networks Based on Hop Distance Statistics

**DOI:** 10.3390/s18010204

**Published:** 2018-01-12

**Authors:** Haejoon Jung, In-Ho Lee

**Affiliations:** 1 Department of Information and Telecommunication Engineering, Incheon National University, Incheon 22012, Korea; haejoonjung@inu.ac.kr; 2 Department of Electrical, Electronic and Control Engineering, Hankyong National University, Anseong 17579, Korea

**Keywords:** Internet-of-Things (IoT), machine-to-machine (M2M) communications, multi-hop networks, hop distance

## Abstract

As an intrinsic part of the Internet of Things (IoT) ecosystem, machine-to-machine (M2M) communications are expected to provide ubiquitous connectivity between machines. Millimeter-wave (mmWave) communication is another promising technology for the future communication systems to alleviate the pressure of scarce spectrum resources. For this reason, in this paper, we consider multi-hop M2M communications, where a machine-type communication (MTC) device with the limited transmit power relays to help other devices using mmWave. To be specific, we focus on hop distance statistics and their impacts on system performances in multi-hop wireless networks (MWNs) with directional antenna arrays in mmWave for M2M communications. Different from microwave systems, in mmWave communications, wireless channel suffers from blockage by obstacles that heavily attenuate line-of-sight signals, which may result in limited per-hop progress in MWNs. We consider two routing strategies aiming at different types of applications and derive the probability distributions of their hop distances. Moreover, we provide their baseline statistics assuming the blockage-free scenario to quantify the impact of blockages. Based on the hop distance analysis, we propose a method to estimate the end-to-end performances (e.g., outage probability, hop count, and transmit energy) of the mmWave MWNs, which provides important insights into mmWave MWN design without time-consuming and repetitive end-to-end simulation.

## 1. Introduction

With the advent of the Internet of Things (IoT), which shifts the paradigm of the Internet from human interconnection into a network of devices, it is predicted that almost 50 billion devices will be connected by 2020 [1]. As a key technology to realize the IoT ecosystem, machine-to-machine (M2M) communication enables wireless devices to constantly interact with each other as well as with their environments without direct human intervention [2,3,4,5,6,7,8]. Furthermore, to enhance network capacity, a fifth generation (5G) cellular system is envisioned to have significantly greater spectrum allocations at millimeter-wave (mmWave) frequency bands. For this reason, in this paper, we investigate multi-hop wireless networks for M2M communications using mmWave.

M2M communication is desirable to interact with a large number of remote devices acting as the interface with end customers, utilities, etc. [5]. For example, various machine-type communication (MTC) devices such as smart meters, signboards, cameras, remote sensors, laptops, and appliances can be interconnected to support wide range of applications. Considering the limited capability of energy harvesting technologies as noted in [9], the subsequent wireless communication techniques must be short-range with low data rates, which makes multi-hop ad hoc networking absolutely necessary in realistic deployment of a large volume of MTC devices [8]. In this paper, we consider M2M communications with low-cost devices such as sensors and low-power mobile machines for applications such as healthcare, energy management, and entertainment [10]. As a related topic, device-to-device (D2D) communication is an emerging technology in cellular networks, where the devices in proximity can directly communicate with each other. From an architectural viewpoint, D2D communication may look similar to multi-hop M2M communication such as mobile ad hoc networks [11]. However, D2D communication is mainly for single hop communications with the involvement of the cellular network in the control plane, and it generally does not inherit multi-hop routing issue in multi-hop wireless networks (MWNs) [12]. We note that such types of D2D communication (single or small number of hops) are out of the scope of this paper. Instead, in this work, we consider multi-hop M2M communications using mmWave.

A fundamental question in MWNs is whether it is advantageous to route over many short hops (short-hop routing) or over a smaller number of longer hops (long-hop routing), as highlighted in [13,14]. In general, the long-hop routing is preferred for delay-sensitive applications, while the short-hop routing is desirable to reduce transmit power consumption [15,16]. For this reason, the hop distance statistics of different routing strategies are extensively studied [13,14,15,16,17,18] in microwave systems. For example, in [13], the hop distance statistics are used to derive the transmit energy consumption assuming Rayleigh fading channel. As in the microwave systems, the routing strategy associated with the hop distance characteristics is crucial in the future generation ad hoc networks using mmWave because the M2M networks with battery-powered devices inherently entail the limited transmission range, which requires multi-hop transmissions [19,20].

To cope with dramatic increase in mobile traffic and extreme device density, the future fifth generation (5G) cellular networks are expected to have mmWave carrier frequency with massive bandwidths and unprecedented number of antennas [21]. Enabled by the availability of a wide spectrum and recent advances in radio frequency integrated circuit (RFIC) technologies, mmWave communication is a key technology to support ever-increasing capacity demand [19,22,23,24]. At mmWave frequency, highly directional transmission using antenna arrays is an effective technique to overcome its heavier path-loss compared to the microwave systems [25,26,27,28]. However, mmWave signals exhibit reduced diffraction and higher susceptibility to blockage compared to microwave signals; thus, mmWave channel is nearly bimodal depending on the presence or absence of line-of-sight (LoS) [21]. For this reason, if the LoS path is blocked by obstacles, an outage event may occur for high data rate applications such as multimedia data transfer that cannot be supported solely by non-LoS paths.

In [29], the authors propose a stochastic model of such blockage effects in mmWave channels, where the probability of the existence of the LoS path is an exponentially decaying function of the distance between two nodes. Using this model, the authors in [30] study the coverage and rate performance in mmWave cellular networks. Moreover, in [31], the signal-to-interference-and-noise-ratio in the mmWave ad hoc network is analyzed in terms of a information theoretical metric (transmission capacity). In this paper, we also assume the blockage model in [29] and focus on the hop distance statistics under the blockage effects. We provide insights to better design multi-hop mmWave M2M communication systems using two representative and generic routing strategies with maximum and minimum possible hop distances, which can be exploited to build routing schemes and higher-layer protocols in the future. The main contributions of this paper are threefold: first, we derive closed form expressions of probability distributions of hop distances for two different routing schemes in the mmWave MWNs unlike the microwave MWNs in [13]; second, we derive the blockage-free hop distance statistics as a baseline to quantify the impact of the blockage effect; third, using the derived hop distance statistics, we estimate end-to-end performances such as outage probability, hop count, and transmit energy, which are compared with simulation results to validate our analysis.

The rest of this paper is organized as follows. The system model is introduced in Section 2. In Section 3, per-hop outage probability and the distance distribution of line-of-sight (LoS) links are derived in the presence of blockage effects. In Section 4, we introduce two routing strategies targeting different system requirements and analyze their hop distance statistics. In Section 5, we propose a method to estimate the end-to-end system performances (e.g., outage probability, hop count, and transmit energy) of mmWave MWNs based on the per-hop statistics. The blockage-free performances are analyzed as baseline cases to quantify the impact of the blockage effects in Section 6. Numerical results are presented in Section 7 to validate our analysis with simulation results. Conclusions are provided in Section 8.

## 2. System Model

**Figure 1:**
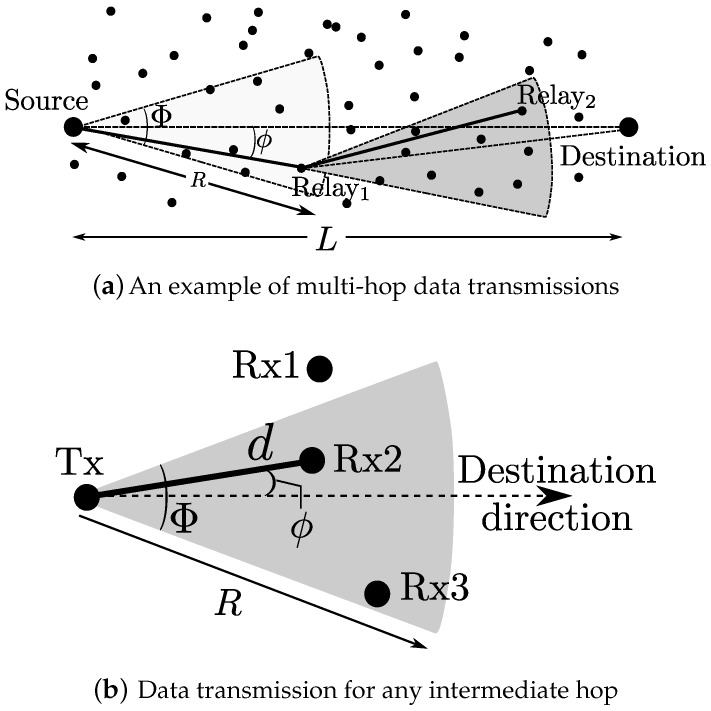
System model.

Stochastic geometry is a powerful tool to model and analyze wireless networks assuming that the locations of nodes or the network structure are random in nature because of their unpredictable spatial characteristics [32]. Poisson point process (PPP), where the number of points (nodes) inside any compact set is a Poisson random variable and the points are uniformly distributed in the compact set, is the most widely used spatial model for networks with uniform node density such as ad hoc networks [33,34,35,36,37] and cellular networks [38]. For this reason, we also assume PPP for the spatial distribution of MTC devices. To be specific, we consider a multi-hop M2M network using mm-wave as shown in Figure 1a, where nodes (devices), which are indicated by the black dots, are uniformly scattered according to a two-dimensional of intensity λ.

Let *N* be the number of nodes that exist in an area A. The probability mass function (PMF) of *N* follows the Poisson distribution as

(1) $$\mathrm{Pr}\left[N=n\right]=e^{-(\lambda A)}\frac{{\left(\lambda A\right)}^{n}}{n!},$$

where Pr[·] denotes the probability of a certain event. For data communications, we assume that all the nodes have the same maximum transmission range of *R*, which is a function of transmit power, antenna gain, and path loss. In addition, as in [31,39], for the analytical tractability, the antenna pattern of the devices is approximated by a sectored model, which has the same gain within its beamwidth Φ (i.e., $\left|\phi \right|\leq \frac{\Phi }{2}$) and zero gain outside as indicated by the gray area in Figure 1b.

Each device is equipped with an electronically steerable directional antenna. Before the route construction, a node operates with Φ=2π to receive short control packets for route construction and periodic beacon, by which each node knows the locations of their neighbors. For example, in the Ad hoc On-Demand Distance Vector (AODV) routing protocol [40], a periodic beacon called Hello message is used to detect each other in their neighborhoods. In general, such control packets are designed to be more easily decoded compared to data packets by using different code rates, modulation techniques, transmit power levels, and packet sizes. Thus, even without beamforming, the transmission range in the neighbor discovery phase can have the same or even extended transmission range compared to the data transmission phase [41].

Similar to [13], in the routing schemes that we consider, a node transmits a packet to one of its blockage-free neighbors that lie within a sector r≤R and $\left|\phi \right|\leq \frac{\Phi }{2}$, where ϕ is with respect to the transmitting node-destination axis as in Figure 1b, assuming all the nodes know the location of the destination, which may be conveyed in a route request packet from the source (e.g., a route request (RREQ) from the source in AODV [40]). In other words, for route construction, the source steers its beam towards the destination and selects one node as a relay following certain criteria, which will be discussed in Section 4. Then, the selected relay also steers its beam towards the destination and recruits its next-hop node within a sector Φ toward the destination in the same manner. Thus, the route built in this way is a zig-zag path, the efficiency of which will be further discussed in Section 5. For the data transmission after the route construction, the nodes in the route steer the beam to their previous-hop and next-hop nodes to receive and forward data packets, respectively. As in AODV [40], we assume that there exists only a single pair of transmitter and receiver in each hop. In other words, the source or a relay can only transmit or forward the data packet to a single node (either the next-hop relay or the destination).

As in [42], with the same beamwidth for transmission and reception, the maximum transmission range *R* in the data transmission phase is given by

(2) $$R=R_{0}{\left(\frac{2\pi }{\Phi }\right)}^{2/\alpha },$$

where α is the path loss exponent and $R_{0}$ is the reference transmission range depending on the transmit power with Φ=2π. We assume that all the nodes operate with the same maximum transmit power and beamwidth. We make this assumption for analytical simplicity to gain design insights, as in [31,42]. However, in practice, MTC devices may have different transmission ranges and beamwidths. We leave the investigation of mmWave M2M networks consisting of devices with non-homogeneous transmission ranges and beamwidths to future work. In the actual data packet transmission, with the given next-hop node, the source and the relays adapt their transmit power to be just strong enough to reach the next-hop node to save energy consumption, as in [13,43]. Therefore, the short-hop routing takes less transmit energy compared to the long-hop routing. We will investigate the transmit energy in more detail in Section 5.2. Moreover, to reflect the blockage effects in the mmWave systems, we use the blockage model based on the stochastic geometry proposed in [29]. Let $r_{i}$ be the distance from a transmitting node (e.g., source or a relaying node) to Node *i* towards the destination. From [29,30,31], the probability of no blockage to Node *i* is

(3) $$P_{\mathrm{LoS},i}=e^{-\beta r_{i}},$$

where β is the blockage parameter that depends on the density and sizes of obstacles blocking LoS paths from the transmitting node to Node *i*. As in [29,44,45,46,47], we assume that an outage occurs if the LoS path is blocked. In other words, even if Node *i* is within the transmission area A, the link to Node *i* is unavailable with the probability of $1-P_{\mathrm{LoS},i}$.

## 3. Node Distribution with LoS Links

In this section, we investigate the spatial distribution of the nodes with blockage-free LoS paths based on non-homogeneous Poisson process, and consider an arbitrary hop between the source and the destination to derive hop distance statistics.

### 3.1. Non-Homogeneous Poisson Process and Outage

Suppose that there are *N* nodes in the transmission area A. Since the *N* nodes are uniformly distributed in A, when the distance from a transmitter to Node *i* (∈{1,…,N}) is $r_{i}$, its probability density function (PDF) is given by

(4) $$f_{r_{i}}\left(x\right)=\frac{2x}{R^{2}},$$

where 0≤x≤R. We define a Bernoulli random variable that indicates the existence of an unblocked LoS path between the transmitter to Node *i*:

(5) $$U_{i}=\left\{\begin{array}{cc} 1, & w.p.\;P_{\mathrm{LoS},i}=e^{\left(-\beta r_{i}\right)},\\ 0, & w.p.\;1-P_{\mathrm{LoS},i}=1-e^{\left(-\beta r_{i}\right)}. \end{array}\right.$$

When $U_{i}=1$, the mmWave link to Node *i* is blockage-free and reliable, and Node *i* is called a *LoS node*. In contrast, $U_{i}=0$ means an outage of the link. We note that the blockage events are assumed to be independent for different links (i.e., $U_{i}$ and $U_{j}$ are independent for i≠j), as in [29,30,31,44,45].

While the spatial distribution of nodes follows the homogeneous Poisson process with intensity λ, the distribution of the LoS nodes is modeled by *non-homogeneous Poisson process* (NHPP) [48] because of the distance-dependent probability function in (5). Based on NHPP, the probability that there are *k* LoS nodes in A is

(6) $$\begin{array}{c} \mathrm{Pr}\left[K=k\right]=\frac{Q^{k}e^{-Q}}{k!}, \end{array}$$

where K≤N for the total (both LoS and NLoS) number of nodes *N* in the area in Equation (1). In addition, *Q* is the intensity of the NHPP, which is a function of *R*, Φ, λ, and β:

(7) $$\begin{array}{c} Q=\int_{0}^{R}\int_{\frac{-\Phi }{2}}^{\frac{\Phi }{2}}\lambda e^{-\beta x}xd\theta dx=\frac{\Phi \lambda \left[1-\left(1+\beta R\right)e^{-\beta R}\right]}{\beta^{2}}. \end{array}$$

The transmitting node experiences an outage, when there is no LoS node (i.e., all $U_{i}$s are zero). Thus, the probability of per-hop outage, where no LoS node exists for a given transmitter, is given by

(8) $$\begin{array}{c} P_{out}=\mathrm{Pr}\left[K=0\right]=e^{-\frac{\Phi \lambda }{\beta^{2}}\left[1-\left(1+\beta R\right)e^{-\beta R}\right]}. \end{array}$$

From the assumption of mutually independent blockage events, as in [29,30,31,44,45], we assume that the outage events in multiple hops are independent to each other.

### 3.2. Distance Distribution of LoS Links

We note that Node *i* becomes the receiver candidate, (i.e., the relaying candidate for the next hop), only if $U_{i}=1$. Thus, let $d_{i}$ be the distance to Node *i* given that the corresponding LoS path is not blocked (i.e., $d_{i}≜r_{i}|U_{i}=1$). Its PDF is then derived as

(9) $$\begin{array}{cc} f_{d_{i}}\left(x\right) & =\frac{\mathrm{Pr}\left[U_{i}=1|r_{i}=x\right]f_{r_{i}}\left(x\right)}{\mathrm{Pr}[U_{i}=1]}=\frac{e^{-\beta x}\cdot \frac{2x}{R^{2}}}{\int_{0}^{R}e^{-\beta x}\cdot \frac{2x}{R^{2}}dx}\\ & =\frac{e^{-\beta x}\cdot \frac{2x}{R^{2}}}{\frac{2\left(1-e^{-\beta R}\left(1+\beta R\right)\right)}{\beta^{2}R^{2}}}=\frac{\beta^{2}xe^{-\beta x}}{1-e^{-\beta R}\left(1+\beta R\right)}, \end{array}$$

where 0≤x≤R. Hence, the cumulative distribution function (CDF) is given by

(10) $$\begin{array}{c} F_{d_{i}}\left(x\right)=\int_{0}^{x}f_{d_{i}}\left(x\right)dx=\frac{e^{\beta (R-x)}\left(-1+e^{\beta x}-\beta x\right)}{e^{\beta R}-1-\beta R}, \end{array}$$

where 0≤x≤R. Based on the probability distributions, the *m*-th moment is

(11) $$\begin{array}{cc} E\{d_{i}^{m}\} & =\int_{0}^{R}x^{m}\frac{\beta^{2}xe^{-\beta x}}{1-e^{-\beta R}\left(1+\beta R\right)}dx\\ & =\frac{e^{\beta R}\left[\Gamma \left(m+2,0\right)-\Gamma \left(m+2,\beta R\right)\right]}{\beta^{m}\left(e^{\beta R}-\beta R-1\right)}, \end{array}$$

where m>−2 and E{·} denotes the expected value. Moreover, $\Gamma \left(a,z\right)=\int_{z}^{x}t^{a-1}e^{-t}dt$ is the incomplete gamma function.

## 4. Routing Strategies and Hop Distance Distributions

**Figure 2:**
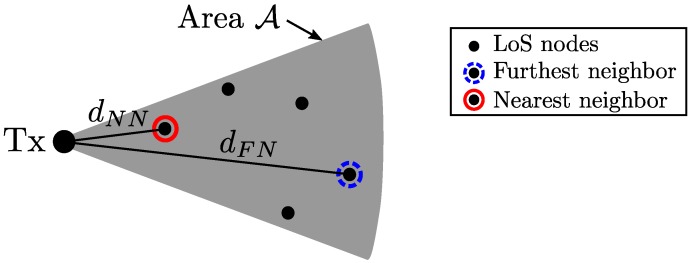
Hop distances of two routing strategies: furthest neighbor (FN) and nearest neighbor (NN).

In this section, we introduce two routing strategies and derive the probability distributions of their hop distances. As the microwave system analysis in [13], to implement the routing schemes, it is assumed that all nodes know their own locations and the source knows the direction towards the destination. Suppose the number of the blockage-free nodes in A is *K*. K=0 corresponds to the outage. On the other hand, if K≥1, we consider two routing schemes, where the next-hop node is the furthest and nearest LoS nodes in its coverage A respectively, as shown in Figure 2. We only consider these two simple but representative routing schemes because this paper is focused on the impact of the blockage effects in mmWave multi-hop M2M communications in terms of hop distance to gain system-level design insights. In practice, the routing protocol should be chosen based on various system requirements such as latency, throughput, reliability, and energy-efficiency. In addition, in case of the M2M networks with selfish nodes, we need consider game theory-based protocol design as in [49,50], which offers incentives to stimulate forwarding. However, it is beyond the scope of this paper.

### 4.1. Furthest Neighbor (FN) Routing

For multi-hop mm-wave transmission, individual hop distance needs to be maximized for delay-sensitive applications such as video and voice traffic. With *K* blockage-free nodes in A, let the hop distance of the furthest neighbor (FN) routing $d_{FN}$ be

(12) $$\begin{array}{c} d_{FN}≜\max\left\{d_{i}\;\mathrm{for}\;1\leq i\leq k|K=k\right\}, \end{array}$$

where $d_{FN}=0$ for K=0. The conditional CDF of $d_{FN}$ given that there is at least one blockage-free node is

(13) $$\begin{array}{cc} F_{d_{FN}|K\geq 1}\left(x\right) & =\mathrm{Pr}\left[d_{FN}\leq x|K\geq 1\right]\\ & \overset{\left(a\right)}{=}\sum_{k=1}^{\infty }\mathrm{Pr}\left[\mathrm{all}\;d_{i}\text{’}s\leq x\;\mathrm{for}\;1\leq i\leq k|K=k\right]\cdot \frac{\mathrm{Pr}[K=k]}{\mathrm{Pr}[K\geq 1]}\\ & \overset{\left(b\right)}{=}\frac{1}{1-P_{out}}\sum_{k=1}^{\infty }{\left(F_{d_{i}}\left(x\right)\right)}^{k}\frac{Q^{k}e^{-Q}}{k!}\\ & =\frac{e^{-Q}\left(e^{Q\cdot F_{d_{i}}\left(x\right)}-1\right)}{1-P_{out}}=\frac{1-e^{\frac{\lambda \Phi e^{-\beta x}\left(-\beta x+e^{\beta x}-1\right)}{\beta^{2}}}}{1-e^{\frac{\lambda \Phi \left(1-e^{-\beta R}\left(\beta R+1\right)\right)}{\beta^{2}}}}, \end{array}$$

where 0<x≤R. In addition, (a) follows from the distribution of the maximum of independent and identically distributed (i.i.d.) random variables as in Equation (12) and Bayes’ theorem [51] to calculate the conditional probability associated with the number of nodes following Poission distribution. In addition, (b) follows from $\mathrm{Pr}\left[K\geq 1\right]=1-P_{out}$, $\mathrm{Pr}\left[K=k\right]=\frac{Q^{k}e^{-Q}}{k!}$, and $\mathrm{Pr}\left[d_{i}\leq x\right]=F_{d_{i}}\left(x\right)$. The corresponding conditional PDF is

(14) $$\begin{array}{cc} f_{d_{FN}|K\geq 1}\left(x\right) & =\frac{dF_{d_{FN}|K\geq 1}\left(x\right)}{dx}\\ & =\frac{\lambda \Phi x\cdot e^{\left[\frac{\lambda \Phi e^{-\beta x}\left(e^{\beta x}-\beta x-1\right)}{\beta^{2}}-\beta x\right]}}{e^{\frac{\lambda \Phi \left[1-e^{-\beta R}\left(\beta R+1\right)\right]}{\beta^{2}}}-1}. \end{array}$$

### 4.2. Nearest Neighbor (NN) Routing

For energy-limited networks (e.g., those with battery-powered devices), the near-distance communication is preferred to save transmit (radiated) energy consumption. In such networks, the nearest neighbor (NN) routing is desirable with the hop distance:

(15) $$\begin{array}{c} d_{NN}≜\min\left\{d_{i}\;\mathrm{for}\;1\leq i\leq k|K=k\right\}. \end{array}$$

As before, $d_{NN}=0$ when K=0. The conditional CDF of $d_{NN}$ for K≥1 is derived as

(16) $$\begin{array}{cc} F_{d_{NN}|K\geq 1}\left(x\right) & =\mathrm{Pr}\left[d_{NN}\leq x|K\geq 1\right]\\ & \overset{\left(a\right)}{=}\sum_{k=1}^{\infty }\left(1-\mathrm{Pr}[\mathrm{all}\;d_{i}\text{’}s>x\;\mathrm{for}\;1\leq i\leq k|K=k]\right)\frac{\mathrm{Pr}[K=k]}{\mathrm{Pr}[K\geq 1]}\\ & \overset{\left(b\right)}{=}\frac{1}{1-P_{out}}\sum_{k=1}^{\infty }\left(1-{\left(1-F_{d_{i}}\left(x\right)\right)}^{k}\frac{Q^{k}e^{-Q}}{k!}\right)\\ & =1+\frac{e^{-Q}\left(e^{Q\cdot (1-F_{d_{i}}\left(x\right))}-1\right)}{1-P_{out}}\\ & =1+\frac{e^{\frac{\lambda \Phi \left(e^{-\beta R}\left(\beta R+1\right)-1\right)}{\beta^{2}}}-e^{\frac{\lambda \Phi \left(e^{-\beta x}\left(\beta x+1\right)-1\right)}{\beta^{2}}}}{1-e^{-\frac{\lambda \Phi \left(1-e^{-\beta R}\left(\beta R+1\right)\right)}{\beta^{2}}}}, \end{array}$$

where 0≤x≤R. Moreover, (a) follows from the distribution of the minimum of i.i.d. random variables as in Equation (15) and Bayes’ theorem to calculate the conditional probability associated with the number of nodes following Poission distribution. In addition, (b) follows from $\mathrm{Pr}\left[K\geq 1\right]=1-P_{out}$, $\mathrm{Pr}\left[K=k\right]=\frac{Q^{k}e^{-Q}}{k!}$, and $\mathrm{Pr}\left[d_{i}>x\right]=1-F_{d_{i}}\left(x\right)$. Hence, the conditional PDF is

(17) $$\begin{array}{cc} f_{d_{NN}|K\geq 1}\left(x\right) & =\frac{dF_{d_{NN}|K\geq 1}\left(x\right)}{dx}\\ & =\frac{\lambda \Phi x\cdot e^{\frac{-\beta^{3}x-\lambda \Phi e^{-\beta R}\left(\beta R+1\right)+\lambda \Phi e^{-\beta x}\left(\beta x+1\right)}{\beta^{2}}}}{e^{\frac{\lambda \Phi [1-e^{-\beta R}\left(\beta R+1\right)]}{\beta^{2}}}-1}. \end{array}$$

**Figure 3:**
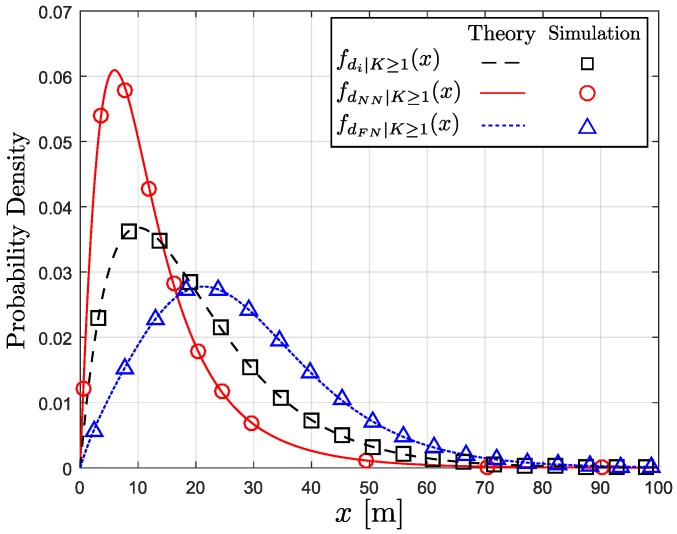
The three conditional probability distribution functions (PDFs); $d_{i}$, $d_{FN}$, and $d_{NN}$.

Figure 3 shows the conditional PDFs in Equations (9), (14), and (17) with λ=0.02, β=0.1, R=100 m, α=2, and $\Phi =60^{\circ }$. In the figure, the analytical curves are consistent with the simulation results. The simulation curves are obtained from $10^{6}$ iterations with random topology. In each random topology realization, the nodes are randomly and uniformly distributed over 1 km^2^ square area, where the number of nodes follows Poisson distribution with the density of λ=0.02 nodes/m^2^. With $10^{6}$ trials, the empirical PDFs of $d_{i}$, $d_{FN}$, and $d_{NN}$ are obtained.

## 5. End-to-End Performance Estimation

In this section, we show how to estimate three key end-to-end performances in mmWave MWNs: *end-to-end hop count*, *outage probability*, and *transmit energy consumption*, based on the hop distance distributions derived in the previous section. To calculate the transmit energy consumption, we follow the model used in [13], where the per-hop transmit (radiation) energy consumption is proportional to $D^{\alpha }$, where *D* is the hop distance and α is the path-loss exponent. We only consider the transmit energy, assuming that the radiation energy to transmit a packet is the most dominant factor in energy consumption of the network as in [13,52]. In fact, the transmission energy consumption is only part of the whole device consumption. Based on the studies on baseband and radio-frequency (RF) circuitries to obtain comprehensive energy consumption models of wireless transceivers, the energy consumption due to the baseband processing circuitry is generally significantly smaller compared to the energy consumption due to RF circuitry [53]. Furthermore, the total transmit energy for the end-to-end communication is the sum of the transmitted energy consumed by the source and all the relays to forward the packets.

### 5.1. Moments of Hop Distance and Average Per-Hop Progress

**Figure 4:**
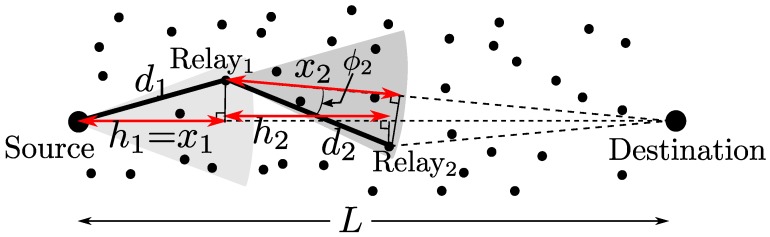
An example illustration of a multi-hop route.

Based on the conditional PDFs of the two routing protocols $f_{d_{FN}|K\geq 1}\left(x\right)$ and $f_{d_{NN}|K\geq 1}\left(x\right)$, we can obtain the conditional *m*-th moment as

(18) $$E\left\{D^{m}|K\geq 1\right\}=\int_{0}^{R}x^{m}\cdot f_{D|K\geq 1}\left(x\right)dx$$

for $D\in \{d_{FN},d_{NN}\}$. m=1 corresponds to the conditional expected hop distance E{D|K≥1}. Then, as in [13], the conditional expected progress per hop, which is indicated by the effective distance travelled along the “Relay_*i*_-Destination” axis for Hop *i* for i≥2 (or the “Source-Destination” axis in the first hop) in Figure 4, is

(19) $$\begin{array}{cc} E\{X|K\geq 1\} & =E\{D\cos\phi |K\geq 1\}\\ & =E\left\{D|K\geq 1\right\}\cdot \int_{-\frac{\Phi }{2}}^{\frac{\Phi }{2}}\cos\phi \cdot \frac{1}{\Phi }\;d\phi \\ & =E\left\{D|K\geq 1\right\}\frac{2}{\Phi }\sin\left(\frac{\Phi }{2}\right). \end{array}$$

This per-hop progress E{X|K≥1} and the α-th moment of the hop distance $E\{D^{\alpha }|K\geq 1\}$, where α is the path-loss exponent, determine key performance metrics in MWNs such as reliability, delay, and transmit energy consumption.

### 5.2. End-to-End Performance Estimates Based on Per-Hop Statistics

In this section, we will investigate how to estimate such end-to-end performances using per-hop statistics such as the *m*-th moments of *D* and $P_{out}$ in the previous sections. First, the average end-to-end hop count η is estimated as

(20) $$\eta \approx \frac{L}{E\{X|K\geq 1\}}=\frac{L\Phi }{2\sin\left(\frac{\Phi }{2}\right)E\left\{D|K\geq 1\right\}},$$

where *L* is the distance between the source and the destination as illustrated in Figure 4. It is noted that *X* is the hop distance *D* projected onto the direction from “*the transmitting node*” to the destination.

Because the actual route is a squiggly line between the source and the destination, this approximation is in fact the lower bound on the actual end-to-end hop count as shown in the example case in Figure 4. Let *H* be the hop distance *D* projected onto the direction from “*the source*” to the destination. In the figure, the progress in the second hop projected onto the transmitter (Relay_1_)-Destination axis $x_{2}=d_{2}\cos\phi_{2}$ is greater than the progress along the source-destination axis, $h_{2}$. Thus, in general, E{X}≥E{H}, which gives $\frac{L}{E\{X\}}\leq \frac{L}{E\{H\}}$ for K≥1. However, as indicated in [13], for a enough large *L*, $x_{i}\approx h_{i}$ for most of hops *i* because the angle between the two axes “Relay_*i*_-Destination” and “Source-Destination” is almost zero, which makes the proposed approximation in Equation (20) becomes tighter.

Using the approximate end-to-end hop count η, the end-to-end outage rate can be estimated as

(21) $$\begin{array}{c} P_{out:EE}\approx 1-{\left(1-P_{out}\right)}^{\eta }=1-{\left(1-P_{out}\right)}^{\frac{L}{E\{X|K\geq 1\}}}\\ =1-{\left[1-e^{-\frac{\Phi \lambda }{\beta^{2}}\left(1-\left(1+\beta R\right)e^{-\beta R}\right)}\right]}^{\frac{L}{E\{X|K\geq 1\}}}, \end{array}$$

where $P_{out}$ is the per-hop outage in Equation (8).

Lastly, the total transmit energy of the multi-hop transmissions normalized by that of the one-hop transmission (i.e., direct transmission from the source to the destination) is approximately given by

(22) $$\begin{array}{cc} Y & \approx \frac{\eta \cdot E\{D^{\alpha }|K\geq 1\}}{L^{\alpha }}=\frac{\Phi E\{D^{\alpha }|K\geq 1\}}{2L^{\alpha -1}\sin\left(\frac{\Phi }{2}\right)E\left\{D|K\geq 1\right\}}, \end{array}$$

α is the path-loss exponent, which is assumed to be two for the LoS condition in the following sections. This normalization is a common way to compare different routing schemes with the common reference by the direct (or one-hop) transmission [54]. Moreover, we note that η and Y are defined only if the end-to-end multi-hop transmission is successful.

It is noted that, if comparing the energy consumptions in the RF circuitry to transmit and receive a packet, the reception energy may be comparable to the transmit energy depending on applications and hardware platforms, as in [55,56]. However, in this paper, following the framework in [13], we do not consider the reception energy. It is expected that the energy consumption for data reception and the corresponding total energy consumption would increase, as the end-to-end hop count increases. However, the exact analysis incorporated with the reception energy consumption will be covered in the future.

## 6. Blockage-Free Scenario

In this section, the blockage-free hop distance statistics are derived. Then, applying the same way to estimate the end-to-end performances in the previous section, analytical expressions for the blockage-free end-to-end performances are obtained to quantify the actual impacts of blockages in the mmWave MWNs.

### 6.1. Blockage-Free Hop Distance

We first derive the conditional *m*-th moments of the two routing schemes for β=0. To derive $E\{d_{FN}|K\geq 1\}$ and $E\{d_{NN}|K\geq 1\}$ in this case, we first find the conditional PDFs for β→0:

(23) $$\begin{array}{cc} \lim_{\beta \to 0}f_{d_{FN}|K\geq 1}\left(x\right) & =\frac{\lambda \Phi xe^{\frac{1}{2}\lambda \Phi x^{2}}}{e^{\frac{1}{2}\lambda \Phi R^{2}}-1}, \end{array}$$

(24) $$\begin{array}{cc} \lim_{\beta \to 0}f_{d_{NN}|K\geq 1}\left(x\right) & =\frac{\lambda \Phi xe^{\frac{1}{2}\lambda \Phi \left(R^{2}-x^{2}\right)}}{e^{\frac{1}{2}\lambda \Phi R^{2}}-1}, \end{array}$$

where 0≤x≤R. Hence, the corresponding *m*-th moments are given by

(25) $$\begin{array}{cc} \lim_{\beta \to 0}E\{ & d_{FN}^{m}\left|K\geq 1\right\}=\frac{2^{m/2}{\left(-\lambda \Phi \right)}^{-\frac{m}{2}}}{e^{\frac{\lambda \Phi R^{2}}{2}}-1}\\ & \cdot \left(\Gamma \left(\frac{m}{2}+1,-\frac{1}{2}\lambda \Phi R^{2}\right)-\Gamma \left(\frac{m}{2}+1,0\right)\right), \end{array}$$

(26) $$\begin{array}{cc} \lim_{\beta \to 0}E\{ & d_{NN}^{m}\left|K\geq 1\right\}=\frac{2^{m/2}{\left(\lambda \Phi \right)}^{-\frac{m}{2}}}{1-e^{-\frac{\lambda \Phi R^{2}}{2}}}\\ & \cdot \left(\Gamma \left(\frac{m}{2}+1,0\right)-\Gamma \left(\frac{m}{2}+1,\frac{1}{2}\lambda \Phi R^{2}\right)\right). \end{array}$$

With these moments, for a given beamwidth Φ, the baseline E{X|K≥1} can be obtained from Equation (19). We note that if λ→∞ on the top of β→0, which corresponds to the high node density and blockage-free environments, $E\left\{d_{FN}^{m}|K\geq 1\right\}\to R^{m}$ and $E\{d_{NN}^{m}|K\geq 1\}\to 0$. These can be readily proved by taking the limit of Equations (25) and (26) as λ→∞.

### 6.2. Blockage-Free End-to-End Performances

Using the same rationale, we can obtain blockage-free end-to-end performance as a baseline. From Equations (20), (25), and (26), the average end-to-end hop counts η for the blockage-free case for FN and NN are obtained as

(27) $$\begin{array}{cc} \eta_{FN}^{*}= & \lim_{\beta \to 0}\frac{L\Phi }{2\sin\left(\frac{\Phi }{2}\right)E\left\{D_{FN}|K\geq 1\right\}}\\ = & \sqrt{-\frac{\lambda \Phi }{2}}\frac{L\Phi \left(e^{\frac{\lambda \Phi R^{2}}{2}}-1\right)}{\sin\left(\frac{\Phi }{2}\right)\left[2\Gamma \left(\frac{3}{2},-\frac{1}{2}\lambda \Phi R^{2}\right)-\sqrt{\pi }\right]}, \end{array}$$

(28) $$\begin{array}{cc} \eta_{NN}^{*}= & \lim_{\beta \to 0}\frac{L\Phi }{2\sin\left(\frac{\Phi }{2}\right)E\left\{D_{NN}|K\geq 1\right\}}\\ = & \sqrt{\frac{\lambda \Phi }{2}}\frac{L\Phi \left(1-e^{-\frac{\lambda \Phi R^{2}}{2}}\right)}{\sin\left(\frac{\Phi }{2}\right)\left[\sqrt{\pi }-2\Gamma \left(\frac{3}{2},\frac{1}{2}\lambda \Phi R^{2}\right)\right],} \end{array}$$

respectively. Hence, the corresponding blockage-free end-to-end outage rate, which can serve as the lower bounds of $P_{out:EE}$ in (21), is given by

(29) $$\begin{array}{c} P_{out:EE}^{*}=\lim_{\beta \to 0}1-{\left(1-P_{out}\right)}^{\eta }\\ =1-{\left(1-\lim_{\beta \to 0}e^{-\frac{\Phi \lambda }{\beta^{2}}\left[1-\left(1+\beta R\right)e^{-\beta R}\right]}\right)}^{\eta^{*}}\\ =1-{\left(1-e^{-\frac{1}{4}\pi \lambda R^{2}}\right)}^{\eta^{*}}, \end{array}$$

where $\eta^{*}\in \left\{\eta_{FN}^{*},\eta_{NN}^{*}\right\}$ in Equations (27) and (28). Furthermore, assuming α=2, the normalized transmit energies of the FN and NN for the end-to-end transmission are

(30) $$Y_{FN}^{*}=\frac{\sqrt{-\frac{2\Phi }{\lambda }}}{L^{\alpha -1}\sin\left(\frac{\Phi }{2}\right)}\frac{1-\Gamma \left(2,-\frac{1}{2}\lambda \Phi R^{2}\right)}{2\Gamma \left(\frac{3}{2},-\frac{1}{2}\lambda \Phi R^{2}\right)-\sqrt{\pi }},$$

(31) $$Y_{NN}^{*}=\frac{\sqrt{\frac{2\Phi }{\lambda }}}{L^{\alpha -1}\sin\left(\frac{\Phi }{2}\right)}\frac{1-\Gamma \left(2,\frac{1}{2}\lambda \Phi R^{2}\right)}{\sqrt{\pi }-2\Gamma \left(\frac{3}{2},\frac{1}{2}\lambda \Phi R^{2}\right).}$$

respectively.

## 7. Numerical and Results

In this section, we validate the analytical results and test the tightness of the end-to-end performance estimation based on per-hop statistics by comparing with simulation results. We assume α=2 to model the LoS channel. In addition, we set $R_{0}=\frac{50}{9}\approx 5.6$ m, which corresponds to the transmission range for the omni-directional antenna Φ=2π. In other words, the beamwidth is $360^{\circ }$, and the maximum possible per-hop transmission range is $R_{0}\approx 5.6$ m assuming battery-powered devices with limited transmission range. Of course, this value of $R_{0}$ can be changed depending on the system parameters and applications. As the beamwidth Φ becomes narrower, the transmission range *R* increases based on Equation (2). The simulation results are averaged over $10^{7}$ iterations with random topology. In each trial, the locations of nodes are uniformly distributed over a 1 km^2^ square region, where the number of nodes follows Poisson distribution with λ=0.015 nodes/m^2^. For a given *L*, the source is located at the center of the 1 km^2^ space. In each hop, the next-hop node is selected following FN or NN. We declare an end-to-end outage of a trial, if an outage occurs in any intermediate hop.

**Figure 5:**
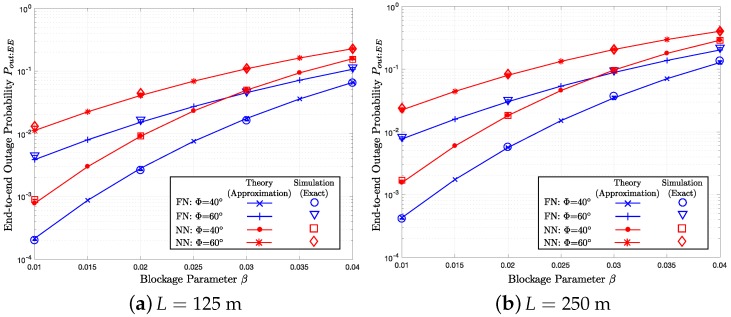
$P_{out:EE}$ versus *β* with m, λ=0.015, $\Phi =\{40^{\circ },60^{\circ }\}$, and R={50 m,33.3 m}.

**Figure 6:**
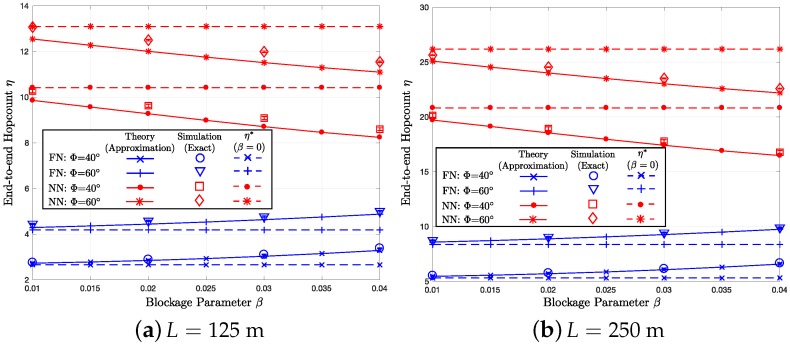
*η* versus *β* with λ=0.015, $\Phi =\{40^{\circ },60^{\circ }\}$, and R={50 m,33.3 m}.

**Figure 7:**
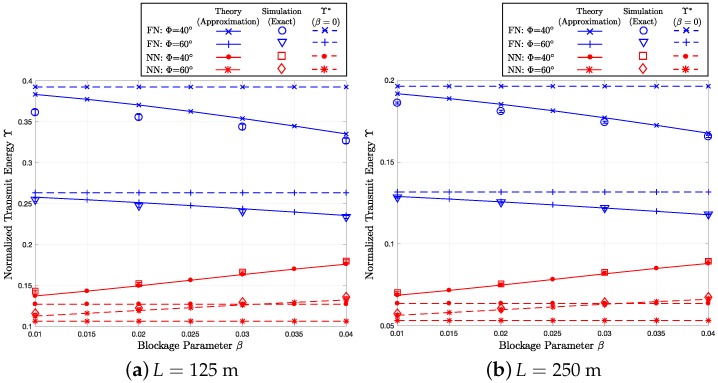
Y versus *β* with λ=0.015, $\Phi =\{40^{\circ },60^{\circ }\}$, and R={50 m,33.3 m}.

Assuming the end-to-end distance *L* of 125 m, Figure 5a, Figure 6a and Figure 7a show how the end-to-end outage probability $P_{out:EE}$, the average end-to-end hop count η, and the total transmit (radiated) energy consumption Y change, respectively, as the blockage parameter varies over 0.01≤β≤0.04. In the figures, we compare the theoretical results (i.e., the approximation proposed in Section 5) with the end-to-end simulation (exact) results for $\Phi =40^{\circ }$ and $60^{\circ }$, which give the transmission ranges of $R=R_{0}\times \frac{360^{\circ }}{40^{\circ }}=50$ m and $R_{0}\times \frac{360^{\circ }}{60^{\circ }}\approx 33.3$ m, respectively. In the three figures, the solid lines with the ‘x’- and ‘+’-markers represent the furthest neighbor (FN) routing with $\Phi =40^{\circ }$ and $60^{\circ }$, respectively. On the other hand, the solid lines with the dot and asterisk markers indicate the nearest neighbor (NN) routing with $\Phi =40^{\circ }$ and $60^{\circ }$, respectively. The circles, triangles, squares, and diamonds correspond to the end-to-end simulation results for FN with $\Phi =40^{\circ }$, FN with $\Phi =60^{\circ }$, NN with $\Phi =40^{\circ }$, and NN with $\Phi =60^{\circ }$, respectively. Moreover, the “I-shape” error bars overlaid on the simulation markers indicate 95% confidence intervals computed using Greenwood’s formula [57]. In Figure 6a and Figure 7a, the baseline cases with β=0, which are indicated by the dotted lines, are illustrated to highlight the impact of the blockage effects. In Figure 5b, Figure 6b and Figure 7b, we perform the same set of simulations for a different end-to-end separation L=250 m and compare the results with the numerical calculations based on the per-hop statistics. To quantify the impact of the blockage on three different performance metrics, the baseline cases without blockage are provided in Table 1, Table 2 and Table 3.

### 7.1. End-to-End Outage Probability $P_{out:EE}$

In Figure 5a,b, as β increases, $P_{out:EE}$ increases. The impact of the blockage can be quantified by comparing with the baseline case $P_{out:EE}^{*}$ in Table 1. Overall, the simulation results are in decent agreement with the theoretical curves, which validates our analysis in Section 5. For a fixed parameter set, FN shows the lower $P_{out:EE}$ compared to NN because FN takes less number of hops to reach the destination, while the outage rate of each hop is the same as defined in Equation (8). For the same reason, $P_{out:EE}$ with L=250 m in Figure 5b is higher compared to L=125 m in Figure 5a.

### 7.2. Average End-to-End Hop Count η

Figure 6a,b display the average end-to-end hop count η versus β graphs. First, FN shows significantly smaller η compared to NN. When β=0.01, both the theory and simulation curves are close to the blockage-free graphs $\eta^{*}$. As β increases, η of FN increases due to less likelihood to find far-distance nodes. On the other hand, the NN shows the opposite trend because of the lower effective density of the LoS nodes in A (less candidates), which results in less chance to find a LoS node with a short hop distance. Hence, the gap between the two routing strategies become smaller, as β increases.

As expected, η with L=250 m is about twice as many as that with 125 m. Moreover, comparing the theoretical and simulation results, the gap between the two is larger for L=125 m compared to L=250 m. This is expected because the error between the analysis and simulation becomes smaller, when the network size grows, as discussed in Section 5.

### 7.3. Average End-to-End Transmit Energy Consumption Y

In Figure 7a,b, we can observe that Y of NN is considerably smaller compared to FN. As β increases, Y decreases for FN, while that for NN increases. Hence, the gap between the two routing schemes becomes smaller, as β increases. In addition, the theory-simulation gap is smaller for L=250 m compared to L=125 m because the error between the two decreases as the end-to-end separation *L* increases.

### 7.4. Impact of Beamwidth Φ

As shown in Figure 5a,b, when the beamwidth Φ decreases from $60^{\circ }$ to $40^{\circ }$, $P_{out:EE}$ decreases both for FN and NN because of the extended maximum hop distance *R* by the antenna gain, which gives smaller end-to-end hop count and higher transmit energy. In addition, in Figure 6a,b, and Figure 7a,b, with the smaller beamwidth $\Phi =40^{\circ }$, η and Y of the two routing schemes become more distinct compared to the wider beamwidth $\Phi =60^{\circ }$ because of the greater R=50 m compared R=33.3 m.

## 8. Conclusions

The hop distance between nodes in wireless multi-hop ad hoc networks have significant impact on the system performance. For this reason, in this paper, we study the hop distance characteristics and their impacts on the end-to-end performances of mmWave multi-hop M2M networks under the blockage effects. We derive the probability distributions of the hop distance with the two routing protocols that have different advantages: FN with the smaller end-to-end delay and NN with the lower transmit energy consumption. In addition, we investigate the blockage-free scenario to establish a baseline to quantify the impact of the blockage effects. Both analysis and simulation indicate that the end-to-end performances of the two routing schemes become more distinct as β decreases or Φ decreases. Based on the hop distance analysis, we estimate the end-to-end performances such as outage, hop count, and transmit energy, which is useful to gain insights into system design guidelines instead of time-consuming simulation. For example, when the blockage effect parameter β is given, a pertinent beamwidth and required transmit energy for FN or NN can be estimated to satisfy a target outage probability. Potential extensions of this paper include addressing a wider scenario with different transmission ranges and beamwidths of MTC devices. In addition, because we only consider the transmit energy in this work, we will investigate more comprehensive analysis on energy consumption with more general energy models. Furthermore, as in [55,56], we will consider a method to maximize the network lifetime by considering the residual energy of battery-powered devices in the network as a long-term plan.

## Figures and Tables

**Table 1:** Blockage-free end-to-end (EE) outage probability $P_{out:EE}$.

Routing	L=125 m		L=250 m	
	$\Phi =40^{\circ }$	$\Phi =60^{\circ }$	$\Phi =40^{\circ }$	$\Phi =60^{\circ }$
FN	$5.5\times 10^{-6}$	$6.8\times 10^{-4}$	$1.1\times 10^{-5}$	$1.4\times 10^{-3}$
NN	$2.2\times 10^{-5}$	$2.1\times 10^{-3}$	$4.3\times 10^{-5}$	$4.2\times 10^{-3}$

**Table 2:** Blockage-free end-to-end (EE) hop count η.

Routing	L=125 m		L=250 m	
	$\Phi =40^{\circ }$	$\Phi =60^{\circ }$	$\Phi =40^{\circ }$	$\Phi =60^{\circ }$
FN	2.657	4.183	5.315	8.367
NN	10.42	13.10	20.83	26.19

**Table 3:** Blockage-free normalized transmit energy consumption Y.

Routing	L=125 m		L=250 m	
	$\Phi =40^{\circ }$	$\Phi =60^{\circ }$	$\Phi =40^{\circ }$	$\Phi =60^{\circ }$
FN	0.392	0.263	0.196	0.132
NN	0.127	0.107	0.064	0.053

## Abbreviations

The following abbreviations are used in this manuscript:

IoT	Internet of things
M2M	Machine-to-machine
Machine-type communication (MTC) D2D	Device-to-device
mmWave	Millimeter-wave
MWN	Multi-hop wireless networks
5G	Fifth generation
RFIC	Radio frequency integrated circuit
LoS	Line-of-sight
NLoS	Non-line-of-sight
PPP	Poisson point process
PMF	Probability mass function
AODV	Ad hoc on-demand distance vector
RREQ	Route request
PDF	Probability density function
NHPP	Non-homogeneous Poisson process
CDF	Cumulative distribution function
FN	Furthest neighbor
NN	Nearest neighbor
EE	End-to-end

## References

[1] Bello O., Zeadally S. (2016). Intelligent device-to-device communication in the internet of things. IEEE Syst. J..

[2] Mumtaz S., Huq K.M.S., Ashraf M.I., Rodriguez J., Monteiro V., Politis C. (2015). Cognitive vehicular communication for 5G. IEEE Commun. Mag..

[3] Palattella M.R., Dohler M., Grieco A., Rizzo G., Torsner J., Engel T., Ladid L. (2016). Internet of Things in the 5G era: Enablers, architecture, and business models. IEEE J. Sel. Areas Commun..

[4] Bangerter B., Talwar S., Arefi R., Stewart K. (2014). Networks and devices for the 5G era. IEEE Commun. Mag..

[5] Zheng K., Hu F., Wang W., Xiang W., Dohler M. (2012). Radio resource allocation in LTE-advanced cellular networks with M2M communications. IEEE Commun. Mag..

[6] Aijaz A., Aghvami A.H. (2015). Cognitive Machine-to-Machine Communications for Internet-of-Things: A Protocol Stack Perspective. IEEE Internet Things J..

[7] Verma P.K., Verma R., Prakash A., Agrawal A., Naik K., Tripathi R., Alsabaan M., Khalifa T., Abdelkader T., Aboghara A. (2016). Machine-to-Machine (M2M) communications: A survey. J. Netw. Comput. Appl..

[8] Chen K.-C., Lien S.-Y. (2014). Machine-to-machine communicatiofns: Technologies and challenges. Ad Hoc Netw..

[9] Vullers R.J.M., Schaihk R.V., Visser H.J., Penders J., Hoof C.V. (2010). Energy Harvesting for Autonomous Wireless Sensor Networks. IEEE Solid State Circuits Mag..

[10] Chen M., Wan J., Gonzalez S., Liao X., Leung V.C.M. (2014). A Survey of Recent Developments in Home M2M Networks. IEEE Commun. Surv. Tutor..

[11] Jung H., Lee I.-H. (2017). Performance analysis of three-dimensional clustered device-to-device networks for Internet of things. Wirel. Commun. Mob. Comput..

[12] Asadi A., Wang Q., Mancuso V. (2014). A Survey on Device-to-Device Communication in Cellular Networks. IEEE Commun. Surv. Tutor..

[13] Haenggi M. (2005). On routing in random Rayleigh fading networks. IEEE Trans. Wirel. Commun..

[14] Haenggi M., Puccinelli D. (2005). Routing in ad hoc networks: A case for long hops. IEEE Commun. Mag..

[15] Jung H., Weitnauer M.A. (2014). Multi-packet opportunistic large array transmission on strip-shaped cooperative routes or networks. IEEE Trans. Wirel. Commun..

[16] Jung H., Weitnauer M.A. Analysis of intra-flow interference in opportunistic large array transmission for strip networks. Proceedings of the 2012 IEEE International Conference on Communications (ICC).

[17] Bettstetter C., Eberspacher J. Hop distances in homogeneous ad hoc networks. Proceedings of the 2003 Vehicular Technology Conference.

[18] Hong X., Xu K., Gerla M. (2002). Scalable routing protocols for mobile ad hoc networks. IEEE Netw..

[19] Qiao J., Shen X.S., Mark J.W., Shen Q., He Y., Lei L. (2015). Enabling device-to-device communications in millimeter-wave 5G cellular networks. IEEE Commun. Mag..

[20] Wang S., Guo W., Zhou Z., Wu Y., Chu X. (2015). Outage probability for multi-hop d2d communications with shortest path routing. IEEE Commun. Lett..

[21] Andrews J.G., Buzzi S., Choi W., Hanly S.V., Lozano A., Soong A.C.K., Zhang J.C. (2014). What will 5G be?. IEEE J. Sel. Areas Commun..

[22] Bogale T.E., Le L.B. (2016). Massive MIMO and mmWave for 5G wireless HetNet: Potential benefits and challenges. IEEE Veh. Technol. Mag..

[23] Boccardi F., Heath R.W., Lozano A., Marzetta T.L., Popovski P. (2014). Five disruptive technology directions for 5G. IEEE Commun. Mag..

[24] Wang C.X., Haider F., Gao X., You X.H., Yang Y., Yuan D., Aggoune H.M., Haas H., Fletcher S., Hepsaydir E. (2014). Cellular architecture and key technologies for 5G wireless communication networks. IEEE Commun. Mag..

[25] Rappaport T.S., Sun S., Mayzus R., Zhao H., Azar Y., Wang K., Wong G.N., Schulz J.K., Samimi M., Gutierrez F. (2013). Millimeter wave mobile communications for 5G cellular: It will work!. IEEE Access.

[26] Hur S., Kim T., Love D.J., Krogmeier J.V., Thomas T.A., Ghosh A. (2013). Millimeter wave beamforming for wireless backhaul and access in small cell networks. IEEE Trans. Commun..

[27] Pi Z., Khan F. (2011). An introduction to millimeter-wave mobile broadband systems. IEEE Commun. Mag..

[28] Ge X., Cheng H., Guizani M., Han T. (2014). 5G wireless backhaul networks: Challenges and research advances. IEEE Netw..

[29] Bai T., Vaze R., Heath R.W. (2014). Analysis of blockage effects on urban cellular networks. IEEE Trans. Wirel. Commun..

[30] Bai T., Heath R.W. (2015). Coverage and rate analysis for millimeter-wave cellular networks. IEEE Trans. Wirel. Commun..

[31] Thornburg A., Bai T., Heath R.W. (2016). Performance analysis of outdoor mmwave ad hoc networks. IEEE Trans. Signal Proc..

[32] Haenggi M., Andrews J.G., Baccelli F., Dousse O., Franceschetti M. (2009). Stochastic geometry and random graphs for the analysis and design of wireless networks. IEEE J. Sel. Areas Commun..

[33] Sousa E.S. (1990). Optimum transmission range in a direct-sequence spread spectrum multihop packet radio network. IEEE J. Sel. Areas Commun..

[34] Hunter A.M., Andrews J.G., Weber S.P. (2008). Transmission capacity of ad hoc networks with spatial diversity. IEEE Trans. Wirel. Commun..

[35] Inaltekin H., Wicker S.B., Chiang M., Poor H.V. (2009). On unbounded path-loss models: Effects of singularity on wireless network performance. IEEE J. Sel. Areas Commun..

[36] Zhang X., Haenggi M. (2012). Random power control in Poisson networks. IEEE Trans. Commun..

[37] Salbaroli E., Zanella A. (2009). Interference analysis in a Poisson field of nodes of finite area. IEEE Trans. Veh. Technol..

[38] ElSawy H., Sultan-Salem A., Alouini M.S., Win M.Z. (2017). Modeling and analysis of cellular networks using stochastic geometry: A tutorial. IEEE Commun. Surv. Tutor..

[39] Mudumbai R., Singh S.K., Madhow U. Medium access control for 60 GHz outdoor mesh networks with highly directional links. Proceedings of the IEEE INFOCOM 2009.

[40] Perkins C.E., Belding-Royer E.M., Das S.R. (2003). Ad Hoc on-Demand Distance Vector (AODV) Routing.

[41] Jakllari G., Krishnamurthy S.V., Faloutsos M., Krishnamurthy P.V., Ercetin O. (2007). A cross-layer framework for exploiting virtual MISO links in mobile ad hoc networks. IEEE Trans. Mob. Comput..

[42] Shokri-Ghadikolaei H., Fischione C. Millimeter wave ad hoc networks: Noise-limited or interference-limited?. Proceedings of the 2015 IEEE Globecom Workshops (GC Wkshps).

[43] Hou T.-C., Li V. (1986). Transmission Range Control in Multihop Packet Radio Networks. IEEE Trans. Wirel. Commun..

[44] Choi J. (2014). On the macro diversity with multiple BSs to mitigate blockage in millimeter-wave communications. IEEE Commun. Lett..

[45] Jung H., Lee I.-H. (2016). Outage analysis of millimeter-wave wireless backhaul in the presence of blockage. IEEE Commun. Lett..

[46] Jung H., Lee I.-H. (2017). Outage analysis of multihop wireless backhaul using millimeter wave under blockage effects. Int. J. Antennas Propag..

[47] Jung H., Lee I.-H. (2016). Connectivity analysis of millimeter-wave device-to-device networks with blockage. Int. J. Antennas Propag..

[48] Browni M. (1972). Statistical Analysis of Non-Homogeneous Poisson Processes.

[49] Ng T.C.-Y., Yu W. (2007). Joint optimization of relay strategies and resource allocations in cooperative cellular networks. IEEE J. Sel. Areas Commun..

[50] Wang B., Han Z., Liu K.J.R. Distributed Relay Selection and Power Control for Multiuser Cooperative Communication Networks Using Buyer/Seller Game. Proceedings of the IEEE INFOCOM 2007 26th IEEE International Conference on Computer Communications.

[51] Crofton M. (1885). Probability, in Encyclopedia Britannica.

[52] Choi J., Ha J., Jeon H. (2013). On the Energy Delay Tradeoff of HARQ-IR in Wireless Multiuser Systems. IEEE Trans. Commun..

[53] Al-Kanj L., Dawy Z., Yaacoub E. (2013). Energy-Aware Cooperative Content Distribution over Wireless Networks: Design Alternatives and Implementation Aspects. IEEE Commun. Surv. Tutor..

[54] Herhold P., Zimmermann E., Fettweis G. (2005). Cooperative multi-hop transmission in wireless networks. Comput. Netw..

[55] Guntupalli L., Martinez-Bauset J., Li F.Y., Weitnauer M.A. (2017). Aggregated Packet Transmission in Duty-Cycled WSNs: Modeling and Performance Evaluation. IEEE Trans. Veh. Technol..

[56] Jung J.W., Weitnauer M.A. (2013). On Using Cooperative Routing for Lifetime Optimization of Multi-Hop Wireless Sensor Networks: Analysis and Guidelines. IEEE Trans. Commun..

[57] Owen A.B. (2004). Empirical Likelihood.

